# Supplementary material for: The Combined Treatment of Curcumin with Verapamil Ameliorates the Cardiovascular Pathology in a Williams–Beuren Syndrome Mouse Model
Source: Int J Mol Sci. 2023 Feb 7;24(4):3261. doi: 10.3390/ijms24043261 (PMC9961051; doi:10.3390/ijms24043261)
Supplement: Supplementary file 1 [file ijms-24-03261-s001.zip › ijms-2189903-supplementary.pdf]

Supplemental Table S1: Statistical data of SBP and BPM

Three-way ANOVA Sex effect SBP	F (DFn, DFd)	P value
Treatment	F (3, 123) = 4.692	P=0.0039
Genotype	F (1, 123) = 57.05	P<0.0001
Sex	F (1, 123) = 3.103	P=0.0807
Treatment x Genotype	F (3, 123) = 3.819	P=0.0117
Treatment x Sex	F (3, 123) = 0.6550	P=0.5813
Genotype x Sex	F (1, 123) = 0.9970	P=0.3200
Treatment x Genotype x Sex	F (3, 123) = 0.1893	P=0.9035

Three-way ANOVA sex effect BPM	F (DFn, DFd)	P value
Treatment	F (3, 125) = 5.543	P=0.0013
Genotype	F (1, 125) = 19.71	P<0.0001
Sex	F (1, 125) = 0.4535	P=0.5019
Treatment x Genotype	F (3, 125) = 1.623	P=0.1873
Treatment x Sex	F (3, 125) = 0,2480	P=0.8627
Genotype x Sex	F (1, 125) = 1.456	P=0.2299
Treatment x Genotype x Sex	F (3, 125) = 0.5289	P=0.6632

Two-way ANOVA SBP	F (DFn, DFd)	P value
Interaction	F (3, 131) = 4.032	P=0.0088
Treatment	F (3, 131) = 5.777	P=0.0010
Genotype	F (1, 131) = 62.25	P<0.0001

Tukey's multiple comparisons test	Adjusted P Value
**VEHICLE:WT vs. VEHICLE:CD**	P<0.0001
VEHICLE:WT vs. VER:WT	P>0.9999
**VEHICLE:WT vs. VER:CD**	P=0.0003
VEHICLE:WT vs. CUR:WT	P>0.9999
**VEHICLE:WT vs. CUR:CD**	P=0.0013
VEHICLE:WT vs. CURVER:WT	P>0.9997
VEHICLE:WT vs. CURVER:CD	P>0.9995
VEHICLE:CD vs. VER:WT	P<0.0001
VEHICLE:CD vs. VER:CD	P=0.9381
VEHICLE:CD vs. CUR:WT	P<0.0001
VEHICLE:CD vs. CUR:CD	P=0.6030

VEHICLE:CD vs. CURVER:WT	P<0.0001
**VEHICLE:CD vs. CURVER:CD**	P<0.0001

**Two-way ANOVA BPM**	**F (DFn, DFd)**	**P value**
Interaction	F (3, 133) = 1,692	P=0,1718
Treatment	F (3, 133) = 5,677	P=0,0011
Genotype	F (1, 133) = 21,49	P<0,0001

Supplemental Table S2: Statistical data of Ascending Aorta parameters

**ANOVA wall thickness T. Media**	**F (DFn, DFd)**	**P value**
Interaction	F (1, 30) = 11,60	P=0,0019
Treatment	F (1, 30) = 9,898	P=0,0037
Genotype	F (1, 30) = 27,99	P<0,0001
**Tukey's multiple comparisons test**	**Adjusted P Value**	
**VEHICLE:WT vs. VEHICLE:CD**	P<0,0001	
VEHICLE:WT vs. CURVER:CD	P=0,4408	
**VEHICLE:CD vs. CURVER:CD**	P=0,0004	

**Two-way ANOVA diameter Lumen**	**F (DFn, DFd)**	**P value**
Interaction	F (1, 30) = 1,262	P=0,2701
Treatment	F (1, 30) = 19,34	P=0,0001
Genotype	F (1, 30) = 42,17	P<0,0001

**Two-way ANOVA ELN content**	**F (DFn, DFd)**	**P value**
Interaction	F (1, 30) = 0,05941	P=0,8091
Treatment	F (1, 30) = 0,5833	P=0,4510
Genotype	F (1, 30) = 43,72	P<0,0001

**Two-way ANOVA Density VSMC**	**F (DFn, DFd)**	**P value**
Interaction	F (1, 20) = 0,1171	P=0,7358
Treatment	F (1, 20) = 9,485	P=0,0059
Genotype	F (1, 20) = 53,93	P<0,0001

**Two-way ANOVA Total Collagen**	**F (DFn, DFd)**	**P value**
Interaction	F (1, 45) = 20,81	P<0,0001
Treatment	F (1, 45) = 2,773	P=0,1028

Genotype	F (1, 45) = 4,878	P=0,0323
**Tukey's multiple comparisons test**	**Adjusted P Value**	
VEHICLE:WT vs. VEHICLE:CD	P<0,0001	
VEHICLE:WT vs. CURVER:CD	P=0,9769	
VEHICLE:CD vs. CURVER:CD	P=0,0005	

**Two-way ANOVA Green Collagen**	**F (DFn, DFd)**	**P value**
Interaction	F (1, 48) = 19,20	P<0,0001
Treatment	F (1, 48) = 2,121	P=0,1518
Genotype	F (1, 48) = 17,77	P=0,0001
**Tukey's multiple comparisons test**	**Adjusted P Value**	
VEHICLE:WT vs. VEHICLE:CD	P<0,0001	
VEHICLE:WT vs. CURVER:CD	P=0,1822	
VEHICLE:CD vs. CURVER:CD	P=0,0015	

**Two-way ANOVA Red Collagen**	**F (DFn, DFd)**	**P value**
Interaction	F (1, 46) = 8,411	P=0,0057
Treatment	F (1, 46) = 0,4011	P=0,5296
Genotype	F (1, 46) = 0,3175	P=0,5759
**Tukey's multiple comparisons test**	**Adjusted P Value**	
VEHICLE:WT vs. VEHICLE:CD	P=0,2564	
VEHICLE:WT vs. CURVER:CD	P=0,8036	
VEHICLE:CD vs. CURVER:CD	P=0,0853	

Supplemental Table S3: Statistical data of Hearts

**Three way ANOVA Sex effect**	**F (DFn, DFd)**	**P value**
Treatment	F (1, 97) = 22,70	P<0,0001
Genotype	F (1, 97) = 15,48	P=0,0002
Sex	F (1, 97) = 0,1639	P=0,6865
Treatment x Genotype	F (1, 97) = 11,97	P=0,0008
Treatment x Sex	F (1, 97) = 6,940	P=0,0098
Genotype x Sex	F (1, 97) = 0,07919	P=0,7790
**Treatment x Genotype x Sex**	**F (1, 97) = 6,555**	**P=0,0120**

**Šídák's multiple comparisons test**	**Adjusted P Value**
VEHICLE:WT-MALES vs. VEHICLE:WT-FEMALES	P=0,0777
VEHICLE:CD-MALES vs. VEHICLE:CD-FEMALES	P>0,9999
CURVER:WT-MALES vs. CURVER:WT-FEMALES	P=0,1547
CURVER:CD-MALES vs. CURVER:CD-FEMALES	P>0,9999

**Two-way ANOVA Heart vs Body weight**	**F (DFn, DFd)**	**P value**
Interaction	F (1, 101) = 20,76	P<0,0001
Treatment	F (1, 101) = 15,13	P=0,0002
Genotype	F (1, 101) = 16,37	P=0,0001

**Tukey’s multiple comparisons test**	**Adjusted P Value**
VEHICLE:WT vs. VEHICLE:CD	P <0,0001
VEHICLE:WT vs. CURVER:WT	P =0,9640
VEHICLE:WT vs. CURVER:CD	P =0,9995
VEHICLE:CD vs. CURVER:CD	P <0,0001

**Two-way ANOVA Thickness LV**	**F (DFn, DFd)**	**P value**
Interaction	F (1, 25) = 7,988	P=0,0091
Treatment	F (1, 25) = 3,832	P=0,0615
Genotype	F (1, 25) = 14,30	P=0,0009

**Tukey’s multiple comparisons test**	**Adjusted P Value**
VEHICLE:WT vs. VEHICLE:CD	P= 0,0009
VEHICLE:WT vs. CURVER:WT	P=0,9306
VEHICLE:WT vs. CURVER:CD	P= 0,5941
VEHICLE:CD vs. CURVER:CD	P= 0,0099

Supplemental Table S4: Statistical data of 3-NT and XOR

**Two-way ANOVA 3-NT Aorta**	**F (DFn, DFd)**	**P value**
Interaction	F (1, 28) = 12,12	P=0,0017
Treatment	F (1, 28) = 11,27	P=0,0023
Genotype	F (1, 28) = 7,793	P=0,0093
**Tukey's multiple comparisons test**	**Adjusted P Value**	
VEHICLE:WT vs. VEHICLE:CD	P=0,0008	
VEHICLE:WT vs. CURVER:WT	P=0,9997	
VEHICLE:WT vs. CURVER:CD	P=0,9756	
VEHICLE:CD vs. CURVER:CD	P =0.0007	

**ANOVA table XOR Aorta (Inmunofluorescence)**	**F (DFn, DFd)**	**P value**
Interaction	F (1, 19) = 5,952	P=0,0247
Treatment	F (1, 19) = 11,49	P=0,0031
Genotype	F (1, 19) = 2,167	P=0,1574
**Tukey's multiple comparisons test**	**Adjusted P Value**	
VEHICLE:WT vs. VEHICLE:CD	P=0,0478	
VEHICLE:WT vs. CURVER:WT	P=0,9002	
VEHICLE:WT vs. CURVER:CD	P=0,5591	
VEHICLE:CD vs. CURVER:CD	P=0,0037	
**One-way ANOVA XOR Aorta (Western)**	**F (DFn, DFd)**	**P value**
Treatment	F (2, 21) = 34,02	P<0,0001
**Tukey's multiple comparisons test**	**Adjusted P Value**	
VEHICLE:WT vs. VEHICLE:CD	P=0,0014	
VEHICLE:WT vs. CURVER:CD	P=0,5770	
VEHICLE:CD vs. CURVER:CD	P=0,0004	

**Two-way ANOVA 3-NT LV**	**F (DFn, DFd)**	**P value**
Interaction	F (1, 36) = 39,09	P<0,0001
Treatment	F (1, 36) = 13,10	P=0,0009
Genotype	F (1, 36) = 29,96	P<0,0001
**Tukey's multiple comparisons test**	**Adjusted P Value**	
VEHICLE:WT vs. VEHICLE:CD	P<0,0001	
VEHICLE:WT vs. CURVER:WT	P=0,2959	

VEHICLE:WT vs. CURVER:CD	P=0,5088
VEHICLE:CD vs. CURVER:CD	P <0,0001

**Two-way ANOVA XOR LV (Inmunofluorescence)**	**F (DFn, DFd)**	**P value**
Interaction	F (1, 16) = 2,697	P=0,1201
Treatment	F (1, 16) = 8,725	P=0,0093
Genotype	F (1, 16) = 8,653	P=0,0096
**One-way ANOVA XOR LV (Western)**	**F (DFn, DFd)**	**P value**
Treatment	F (2, 21) = 34,02	P<0,0001
**Tukey's multiple comparisons test**	**Adjusted P Value**	
VEHICLE:WT vs. VEHICLE:CD	P<0,0001	
VEHICLE:WT vs. CURVER:CD	P=0,0145	
VEHICLE:CD vs. CURVER:CD	P=0,0001	

Supplemental Table S5: Statistical data of pNRF2

**Two-way ANOVA Aorta**	**F (DFn, DFd)**	**P value**
Interaction	F (1, 34) = 19,30	P=0,0001
Treatment	F (1, 34) = 17,89	P=0,0002
Genotype	F (1, 34) = 36,27	P<0,0001
**Tukey's multiple comparisons test**	**Adjusted P Value**	
VEHICLE:WT vs. VEHICLE:CD	P<0,0001	
VEHICLE:WT vs. CURVER:WT	P=0,9995	
VEHICLE:WT vs. CURVER:CD	P=0,5890	
VEHICLE:CD vs. CURVER:CD	P <0,0001	
**One Way ANOVA Heart**	**F (DFn, DFd)**	**P value**
Treatment	F (2,28) = 8	P=0.0014
**Tukey's multiple comparisons test**	**Adjusted P Value**	
VEHICLE WT vs. VEHICLE CD	P=0.0084	
VEHICLE WT vs. CURVER CD	P=0.8771	
VEHICLE CD vs. CURVER CD	P=0.002	

Supplemental Table S6: Statistical data of treatment intake and body weight

Two-way RM ANOVA Intake-MALES	F (DFn, DFd)	P value
Treatment x Time	F (45, 240) = 0,7963	P=0,8189
Treatment	F (3, 16) = 0,2570	P=0,8552
Time	F (6,238, 99,80) = 0,6699	P=0,6799

Two-way RM ANOVA Intake-FEMALES	F (DFn, DFd)	P value
Treatment x Time	F (45, 240) = 0,7335	P=0,8936
Treatment	F (3, 16) = 1,846	P=0,1794
Time	F (6,061, 96,97) = 0,9496	P=0,4642

Two-way ANOVA Intake- WT vs CD	F (DFn, DFd)	P value
Interaction	F (3, 24) = 0,1347	P=0,9384
Treatment	F (3, 24) = 0,5263	P=0,6685
Genotype	F (1, 24) = 2,915	P=0,1007

Two-way ANOVA Body weight- MALES	F (DFn, DFd)	P value
Interaction	F (3, 102) = 0,9029	P=0,4425
Treatment	F (3, 102) = 1,219	P=0,3068
Genotype	F (1, 102) = 92,76	P<0,0001

Two-way ANOVA Body weight- FEMALES	F (DFn, DFd)	P value
Interaction	F (3, 62) = 1,681	P=0,1803
Treatment	F (3, 62) = 1,483	P=0,2279
Genotype	F (1, 62) = 38,57	P<0,0001

Supplemental Figure

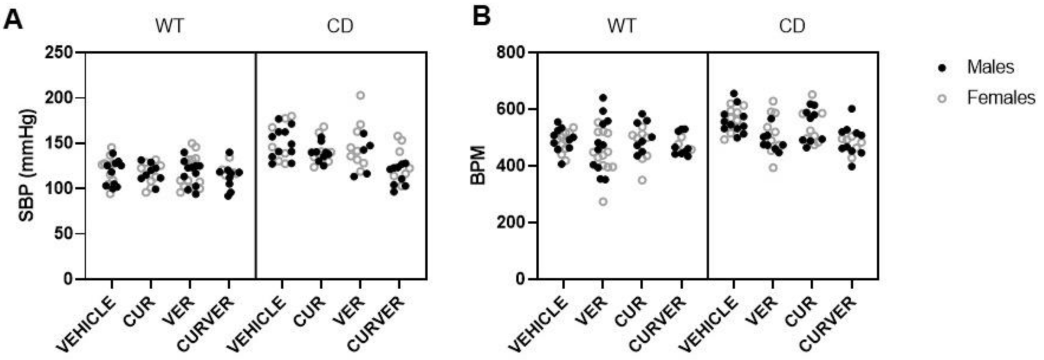

Figure S1: No effect of sex on SBP and BPM

(A) SBP and **(B)** BPM measurements in the conscious state using the indirect tail-cuff method in WT (n=14-23) and CD (n=14-18) mice treated with single (CUR, VER) or combined (CURVER) treatments. No significant differences were observed due to sex. Data are represented as mean ± SEM. Three-way ANOVA. Statistical data in Tables S1

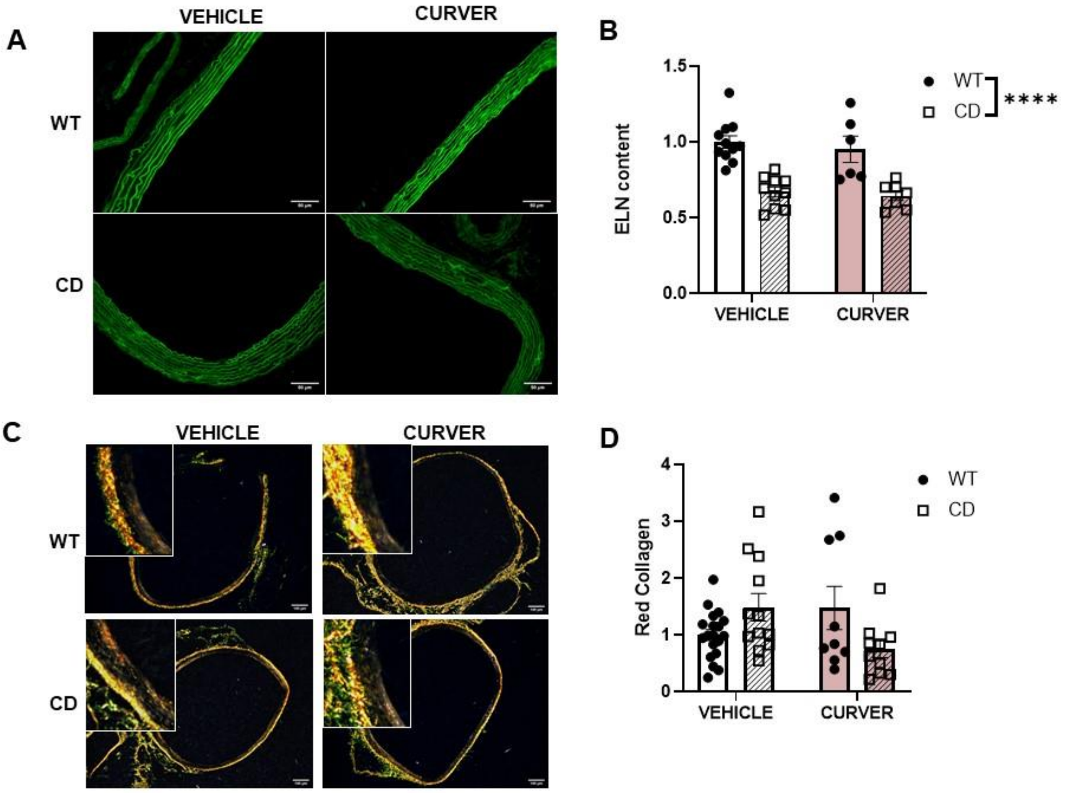

Figure S2: Analysis of ascending aortas

(A) Representative images of green autofluorescence ELN of ascending aortas **(B)** No significant differences were observed in ELN content due to treatment. **(C)** Representative images of ascending aortas under polarised light. A representative magnification is presented in the upper left box. **(D)** No significant differences were

observed in mature (red) collagen content. Data are represented as mean ± SEM. Two-way ANOVA. Statistical data in Tables S2

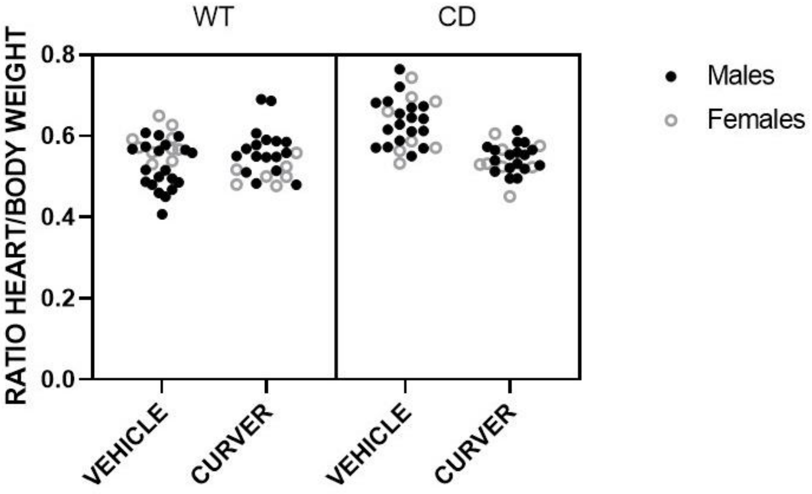

Figure S3: No effect of sex on the heart vs body weight ratio

Data are represented as mean ± SEM. Three-way ANOVA. Statistical data in Table S3

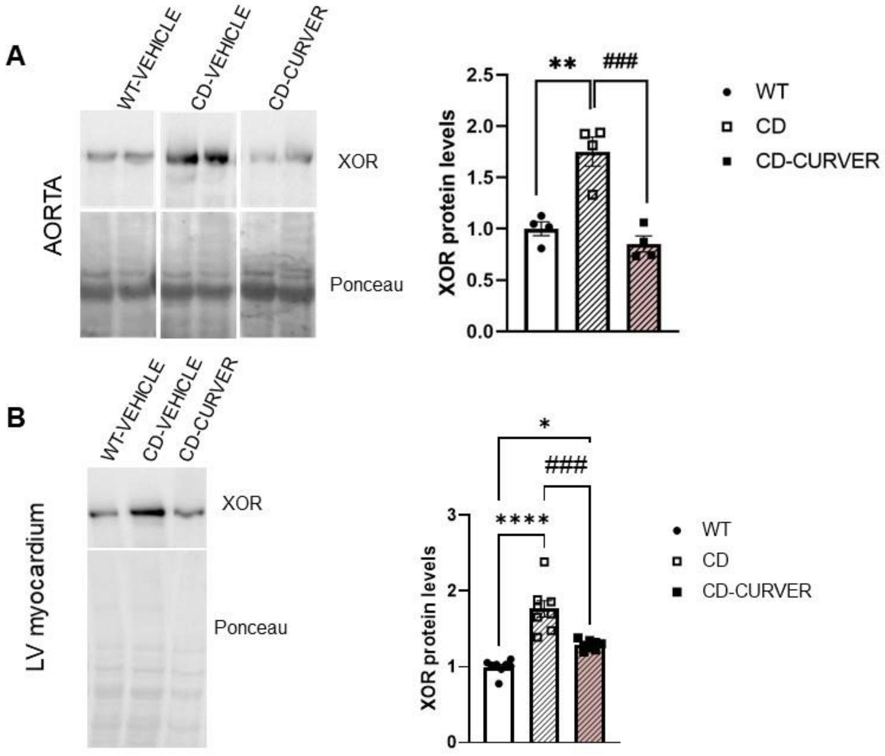

Figure S4: Western blot analysis of XOR levels in ascending aortas and LV-myocardium

Representative images of western blot analysis and the corresponding histogram quantification. Significant difference among groups are shown after one-way ANOVA

followed by Tukey's multiple comparisons test for ascending aorta ($F_{(2,9)}$ =22, p=0.0003) **(A)** and LV-myocardium ($F_{(2,21)}$ =34.02, p<0.0001) **(B)**.

Data are represented as mean ± SEM. * Effect of genotype; #, effect of treatment. ***,### p<0.001; ****,#### p< 0.0001. Statistical data in Table S4

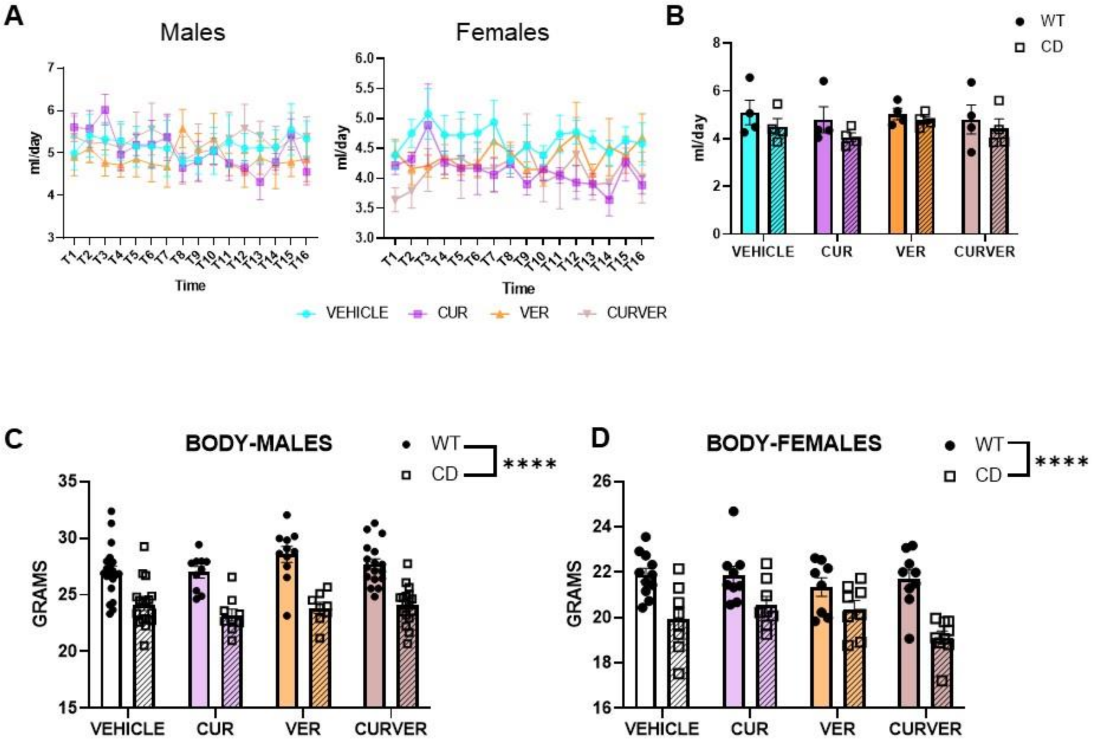

Figure S5: Treatment intake and body weight

(A) The amount of drunk per cage was quantified and normalised to the number of animals per cage (2 to 4) and to the time between each change (48 to 60 hours). Consumption was not significantly different between groups ($F_{45,240}$ = 0.7963, p = 0.8189 in males; $F_{45,240}$ = 0.7335, p = 0.8936 in females). **(B)** Daily intake was not significantly different among WT mice and the other groups ($F_{3,24}$ = 0.134, p = 0.9384). **(C, D).** None of the treatments altered the reduced body weight presented by CD compared with WT mice (effect of genotype $F_{1,102}$ = 92.76, p<0.0001 in males; $F_{1,62}$ = 38.57, p<0.0001 in females).
